# The regulatory role of GABA_A_ receptor in *Actinia equina* nervous system and the possible effect of global ocean acidification

**DOI:** 10.1007/s00424-021-02628-w

**Published:** 2021-10-11

**Authors:** Sergii Snigirov, Sergiy Sylantyev

**Affiliations:** 1 Biological Department, Odesa National Mechnikov University, 2 Shampanskiy Lane, Odesa, 65058 Ukraine; 2 Rowett Institute, University of Aberdeen, Ashgrove Road West, Aberdeen, AB25 2ZD UK

**Keywords:** GABA_A_ receptor, pH-dependent receptor, Diazepam, *Actinia equina*, Global warming, Startle response

## Abstract

Global warming and connected acidification of the world ocean attract a substantial amount of research efforts, in particular in a context of their impact on behaviour and metabolism of marine organisms, such as Cnidaria. Nevertheless, mechanisms underlying Cnidarians’ neural signalling and behaviour and their (possible) alterations due to the world ocean acidification remain poorly understood. Here we researched for the first time modulation of GABA_A_ receptors (GABA_A_Rs) in *Actinia equina* (Cnidaria: Anthozoa) by pH fluctuations within a range predicted by the world ocean acidification scenarios for the next 80–100 years and by selective pharmacological activation. We found that in line with earlier studies on vertebrates, both changes of pH and activation of GABA_A_Rs with a selective allosteric agonist (diazepam) modulate electrical charge transfer through GABA_A_R and the whole-cell excitability. On top of that, diazepam modifies the animal behavioural reaction on startle response. However, despite behavioural reactions displayed by living animals are controlled by GABA_A_Rs, changes of pH do not alter them significantly. Possible mechanisms underlying the species resistance to acidification impact are discussed.

## Introduction

The present rise of atmospheric CO_2_ significantly increases the partial pressure of CO_2_ in the world oceans and, consequently, leads to ocean acidification (OA). Different model scenarios predict a decline in pH values of oceanic waters by up to 0.45 units by the year 2100 [7, 39], with profound consequences to marine ecosystems [14]. In this context, the potential impact of OA on coral cnidarians (Cnidaria: Anthozoa, Scleractinia) attracts understandably significant research efforts given the global role of coral reefs and their sensitivity to water pH and connected fluctuations of carbonate–bicarbonate balance. However, how the OA-driven responses alter the behaviour of the soft-bodied forms of anthozoans and modify their long-term ecological perspectives is to a large extent unknown. During the last decade, several research groups have reported a negligible (or even positive) impact of OA on the metabolism of sea anemones of species supporting microalgal endosymbionts of *Symbiodinium* genus due to rapid changes in endosymbionts’ biochemistry and photosynthesis capacity [16, 20, 40]. Nevertheless, the impact of OA on non-symbiotic sea anemones has yet to be determined.

From the neurophysiological perspective, sea anemones such as *Actinia equina* (*A. equina*) (Cnidaria: Anthozoa, Actiniaria) are “…for all intents and purposes, little more than guts with tentacles” [19]. This definition clearly reflects a limited set of reactions generated by a nervous system of the simplest morphology (nerve net) amongst metazoans [12]. Thus, the factors that could modulate the prominence and features of species’ behavioural profile within such a simple nervous system are of considerable interest. This area has a relatively recent and irregular research history, where the startle response of the non-symbiotic species *A. equina* is used as a quantifying gauge of anemones’ adaptability to climate change and the expression of their personality [5, 29].

GABA_A_-receptors (GABA_A_Rs) are the major inhibitory neurotransmitter receptors in the animal nervous system. These receptors and elements of the synthesis and degradation of their endogenous ligand γ-aminobutyric acid (GABA) have been repeatedly shown to be present in different actinia species [1, 10], whereas the functional signature of GABA_A_Rs was demonstrated in Cnidaria of other classes [17, 32]. The effects of GABA_A_Rs in vertebrates’ neural cells were demonstrated to be pH-dependent due to protonation of the receptors’ extracellular domain. Fluctuations of pH by a few tenths of a unit make a significant impact on neural cell excitability and inter-neuronal signalling [8, 21, 43]. The level of sensitivity to pH fluctuations depends on the GABA_A_R subunit composition [21]. In anthozoans GABA_A_Rs are likely to be of a different subunit composition to that in vertebrates’ cells. Therefore, anthozoans are a prospective object for research of pH-related functionality of GABA_A_Rs and of the impact of environmental pH fluctuations on neural signalling.

The GABA_A_R is a traditional target for the treatment of neural disorders, where the effects of GABA_A_R ligands of the benzodiazepine type (such as diazepam [DZP]) are commonly associated with corrections of individual behaviour and personality profile [24, 41, 42]. However, to the best of our knowledge, to date the functional effects of benzodiazepines in Anthozoa remain unknown.

Hence, in this study, we aimed to test the impact of pH fluctuations within the OA-related interval on the nervous system of *A. equina* at different levels: from a single GABA_A_R to the generation of the startle response by living animals, connecting these points through the functional profile of a separate excitable cell. Additionally, we intended to clarify the DZP impact on anthozoan GABA_A_R functions at different organizational levels (single receptor, single cell, and animal behaviour). Inasmuch sensitivity to environmental factors and bioactive compounds in invertebrates may differ between ecologically and geographically disengaged populations of the same species [26], we aimed to perform experiments on animals representing two populations separated both ecologically and geographically: *A. equina* from both the inter-tidal zone of the Scottish North Sea coast and from the Black Sea wherein tides are absent [2].

The inter-annual fluctuations of pH both in the North Sea and in the Black Sea are within a range of 7.5–8.5 [4, 35]. Therefore, to explore a potential impact of OA (acidification shift by ≤ 0.45 unit [7, 39]) on GABA_A_R-mediated effects and to compare these effects to those observed at pH characteristic to present conditions, we set out to perform experiments at a pH range from 8.5 to 7.0.

## Materials and methods

### Ethical statement

*A. equina* is not protected under either UK Animals (Scientific Procedures) Act 1986 nor listed in the general provisions of the European Directive (2010) on the protection of animals used for scientific purposes. Nevertheless, the study was conducted in accord with the ASAB Guidelines for the treatment of animals in teaching and research [6]. All animals involved into behavioural tests were then released near the point of collection. Environmental parameters in research aquariums were consistently within normal tolerance ranges for corresponding population.

### Behavioural tests

Sea anemones of the North Sea population were collected in a tidal zone of a rocky coast to the north of the town of Dunbar (56° 0′ 11″ N, 2° 31′ 48″ W). Sea anemones of the Black Sea population were collected at a depth of 2–5 m near the shore of Zmiinyi Island (45° 15′ 36″ N, 30° 1′ 12″ E) [37]. A distance of 2–3 m was left between the animals collected, to avoid collecting cloned individuals [11]. Animals were housed in experimental aquaria of 1 m^3^ volume, half-filled (0.5 m^3^) with sea water. Water temperature was held at a level similar to that observed at the time of collection: 12 ± 1 °C for the North Sea animals and 27 ± 1 °C for the Black Sea animals, bubbled with air compressors. Evaporating sea water volumes were replaced with distilled water. Animals were kept at a 12:12-h light/dark cycle and kept for 7 days to acclimatize before the start of the behavioural tests. Animals were fed once per 3 days with small pieces of fresh mussel meat. In behavioural tests, the startle response was induced by rapid ejection of a 10-ml syringe filled with aquarium water into actinia’s mouth opening from 1–3-cm distance. The length of a startle response was registered as a time interval between closure of actinia’s mouth with simultaneous tentacle retraction and subsequent mouth opening with tentacle straightening. In the experiments involving DZP, 100 µM solution of DZP in sea water was used. pH in both in vivo and in vitro experiments was adjusted with HCl and NaOH. Behavioural tests commenced in 1.5–2 h after pH adjustment since after a change of external pH, intracellular pH in actinia cells comes to the normal values in 35–40 min [23].

### Electrophysiology

Electrophysiological recordings were performed on dissociated myoepithelial cells obtained with a protocol proposed by Holman and Anderson [15]. Briefly, after being relaxed and anesthetized in 350 mM MgCl_2_, the animals were dissected. The upper quarter of the actinia stem which neighbours the oral disc was cut into small pieces, then loosened with papain (3.5 mg per ml of sea water) and triturated within a syringe with an 18-gauge needle. Whole-cell patch-clamp recordings were performed in a seawater-perfused chamber at room temperature (~ 22 °C). The intracellular solution for current-clamp recordings contained (in mM) 126 K-gluconate, 8 NaCl, 5 HEPES, 15 glucose, 1 MgSO_4_·7H_2_O, 2 BAPTA, and 3 Mg-ATP, while for voltage-clamp recordings 126 CsCl, 10 KOH-HEPES, 10 BAPTA, 8 NaCl, 5 QX-314, 2 Mg-ATP, and 0.3 GTP (pH 7.2, 295 mOsm). To isolate GABA_A_R response in outside-out patches, we added to perfusion solution 20 µM APV (to block NMDA receptors), 10 µM NBQX (to block AMPA receptors), 50 nM CGP-55845 (to block GABA_B_ receptors), and 1 µM strychnine (to block glycine receptors). Recordings were performed with a Multiclamp-700B amplifier and a Digidata 1550 digitizer, and the recorded traces were digitized at a 10 kHz rate and digitally filtered offline. To apply different perfusion solutions on the same membrane patch in experiments on outside-out patches, the rapid solution exchange system was used [38]. To avoid confusion due to (possible) response modification generated by uncontrolled factors, in different experiments, we used a different order of pH modifications: from lower to higher or from higher to lower values. The single receptor open-time fraction was calculated as *t*_o_/*t*_f_, where *t*_o_ is a time in an open state and *t*_f_ is a full time of recording. Automated detection of the single channel openings was performed with a threshold detection algorithm of Clampfit 11 software package, with a detection threshold set 2.5 pA more negative than a baseline conductance and a minimum event length of 2 ms.

Nonlinear fitting of concentration–response curves was performed with a Hill equation

$$E=\frac{{\left[C\right]}^{{n}_{H}}}{{{K}_{d}}^{{n}_{H}}+{\left[C\right]}^{{n}_{H}}}$$

via a Newton–Raphson iteration method, where *E* is an effect of GABA, *C* is concentration of GABA, *K*_*d*_ is an apparent dissociation constant, and *n*_*H*_ is a Hill coefficient (positive for an increasing function and negative for a decreasing function).

Repeated-measures two-way analysis of variance with Geisser-Greenhouse correction for sphericity and Tukey post-hoc test (RM-ANOVA, performed with GraphPad Prism 8.0 software), nonlinear curve fitting (performed with Wolfram Mathematica 11 package) and Student’s paired t-test (performed with MS Excel) were used as indicated. GABA, QX-314, NBQX, CGP-55845, and diazepam were purchased from Tocris Bioscience, while all other chemicals were purchased from Sigma-Aldrich.

## Results

**Fig. 1:**
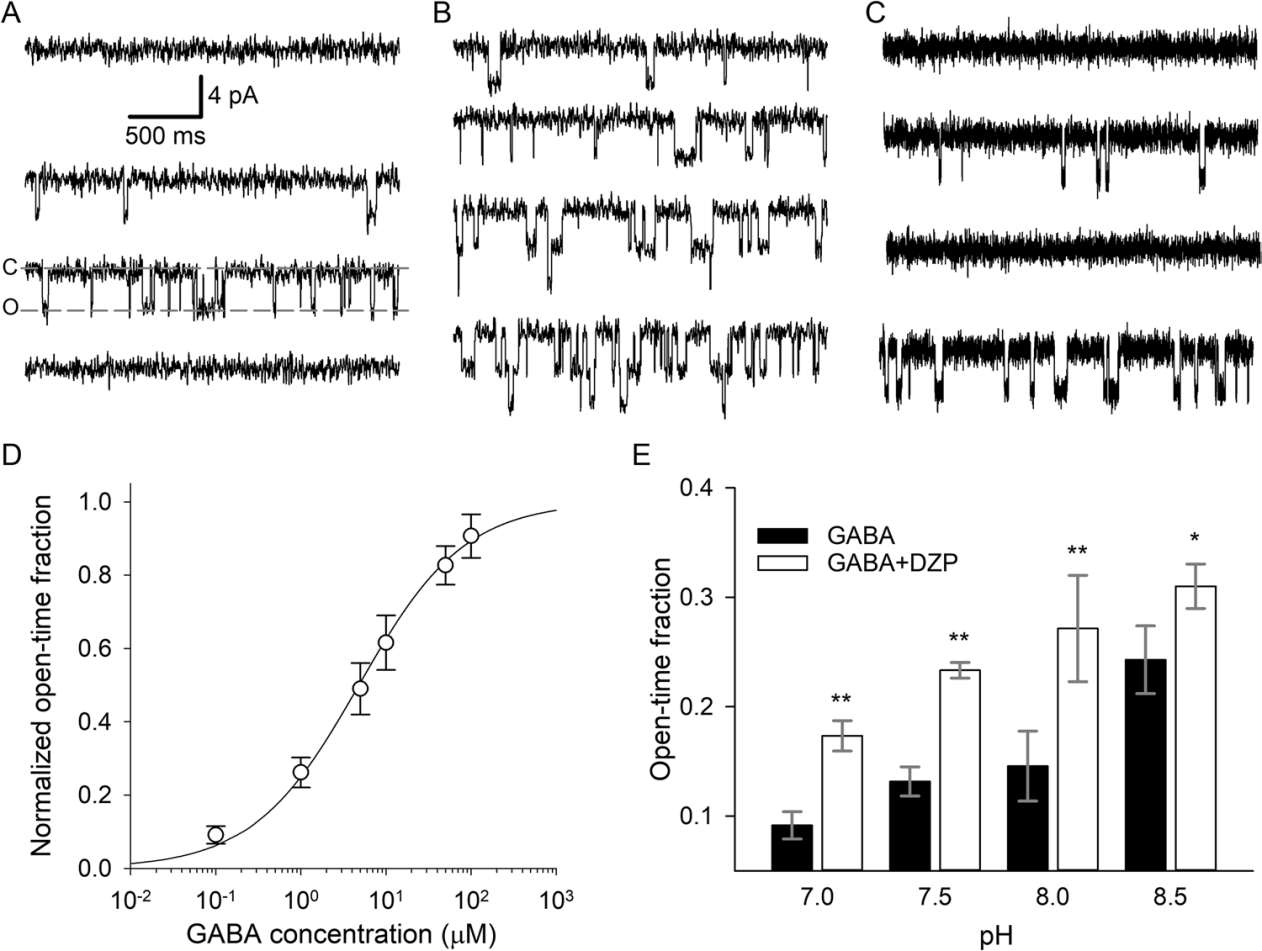
GABA and DZP activate GABA_A_Rs in *A. equina* cell membrane patches. **A**–**C** Example traces recorded consequently from the same membrane patch. **A** Test on GABA_A_R pharmacological specificity. From top to bottom: control (no GABA_A_R ligands), GABA 0.1 µM, MSC 1 µM, MSC 1 µM + PTX 20 µM. “C” and “O” indicate closed and open state of the receptor, respectively. Scale bars apply for **A**–**C**. **B** Ascending concentrations of GABA increase GABA_A_R open-time fraction. From top to bottom: GABA 0.1 µM, 1 µM, 10 µM, 100 µM. **C** DZP impacts GABA_A_R opening frequency only in presence of GABA. From top to bottom: control (no GABA_A_R ligands), GABA 0.1 µM, DZP 100 µM, GABA + DZP. **D** Concentration–response curve generated in **B**. Open-time fraction normalized to that obtained for 1 mM GABA. **E** Statistical summary for **C** at different pH values: pH increase upregulates GABA_A_R activity. Asterisks denote significance of difference from the “GABA only” effect at corresponding pH; * *P* < 0.05, ** *P* < 0.01, Student’s paired t-test, *n* = 6–7 pairs

At the initial stage of this project, we tested whether GABA_A_R functional profile in *A. equina* is pH-dependent and if DZP can modify GABA_A_R activity. To do this, we recorded in voltage-clamp mode GABA_A_R single-channel openings in outside-out membrane patches excised from myoepithelial cells (Fig. 1). Here and in further experiments, we did not observe a significant difference between North Sea and Black Sea actinia for any tested parameter. We thus provide combined data received from animals of both populations.

At first, we tested pharmacological specificity of actinias’ GABA_A_Rs. To do this, we applied different perfusion solutions at the same membrane patch. We found that GABA (100 nM) and a specific GABA_A_R agonist MSC (1 µM) evoke single-channel openings, whereas GABA_A_R open channel blocker PTX (20 µM) shuts GABA_A_R openings induced by MSC (Fig. 1A). Next, we tested the concentration–response effect of GABA on actinias’ GABA_A_Rs (Fig. 1B and D). The concentration–response dependence parameters were fitted as *K*_*d*_ = 4.9 µM and *n*_*H*_ = 0.66. After that, to clarify the effect of DZP, we compared an open-time fraction of recorded trace under control conditions, with 100 nM GABA, 100 µM DZP, and GABA + DZP. Neither under control conditions nor with DZP alone did we register GABA_A_R openings (Fig. 1C). On the contrary, the application of GABA triggered GABA_A_R openings and frequency increased significantly under GABA + DZP (Fig. 1C and E). This experiment was repeated with four pH values: from 7.0 to 8.5 with a 0.5 unit step. To quantify the experimental output, here and in further experiments, we used two-way RM-ANOVA with pharmacological interventions as factor 1 and pH values as factor 2.

RM-ANOVA on the open-time fraction generated the following results. Factor 1: *F*_(1, 22)_ = 65.42, *P* < 0.0001; factor 2: *F*_(3, 22)_ = 5.81, *P* = 0.0044; factor 1 × factor 2: *F*_(3, 22)_ = 1.27, *P* = 0.308. Tukey test on factor 2: *P* = 0.0026 for pH 7.0 vs. pH 8.5, *P* > 0.05 for all other comparisons.

We thus found that DZP alone has no detectable effect on GABA_A_R in *A. equina* but significantly enhances the effect of GABA, as well as that actinia’s GABA_A_R functioning is pH-dependent.

**Fig. 2:**
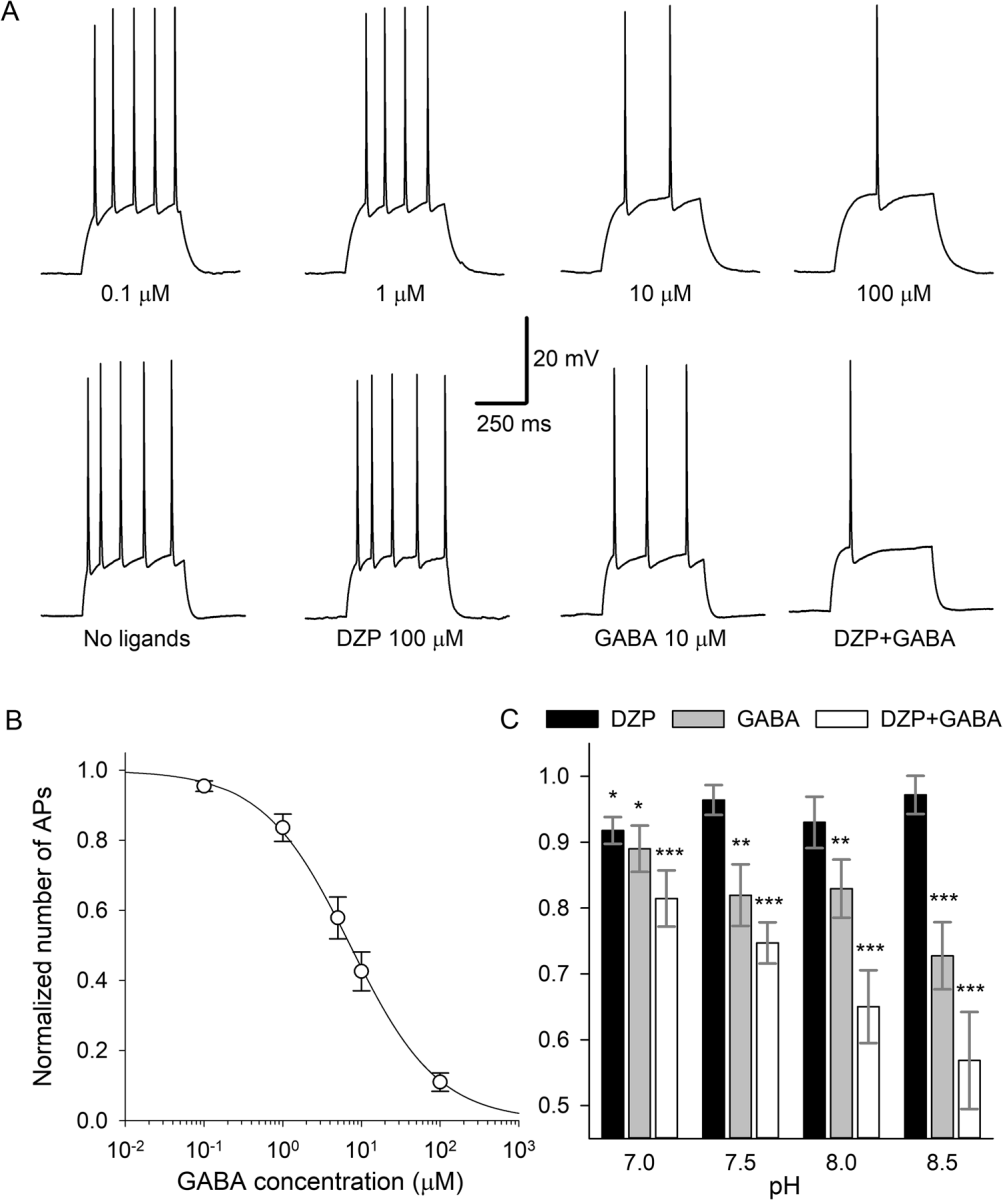
Impact of GABA_A_Rs on action potential generation. **A** Example traces. Application of GABA_A_R agonists reduces the number of APs propagated upon a standard depolarising current injection. Top row: increasing GABA concentrations, recordings from the same cell. Bottom row: application of different GABA_A_R agonists. Scale bars apply to all traces. **B**, **C** Statistics on **A**. **B** concentration–response dependence between applied GABA and a number of APs per standard depolarising current injection; AP numbers are normalized to the value obtained in the same cell with no GABA added. Vertical axis label applies to **B** and **C**. **C** The number of APs generated at different pH values; number of APs obtained under pharmacological interventions normalized to control value (when no GABA_A_R ligands added) obtained in the same cell. Asterisks denote significance of difference from control (unity); * *P* < 0.05, ** *P* < 0.01, *** *P* < 0.001, Student’s paired t-test, *n* = 9–10 pairs

Next, we tested the impact of GABA_A_R on intercellular signalling. To do this, we performed a whole-cell current-clamp recording of action potential (AP) generation in myoepithelial cells. In this experiment, we delivered standard 500 ms depolarising current injections, monitoring the modulation of the AP generation frequency caused by GABA_A_R ligands (GABA, DZP, GABA + DZP) and pH (Fig. 2). We found that pharmacological interventions significantly decrease the number of APs generated upon depolarising current injection, and this effect changes quantitatively under different pH levels.

RM-ANOVA on a number of APs. Factor 1: *F*_(1.95, 66.28)_ = 63.97, *P* < 0.0001; factor 2: *F*_(3, 34)_ = 3.1, *P* = 0.0395; factor 1 × factor 2: *F*_(9, 102)_ = 3.11, *P* = 0.0024. Tukey test on factor 1: *P* < 0.0001 for all comparisons; on factor 2: *P* > 0.05 for all comparisons. On top of that, to obtain more detailed characteristic of the GABA role in intercellular signalling, we next generated a concentration–response dependence for GABA’s modulatory impact on AP generation (Fig. 2). Here nonlinear fitting yielded *K*_*d*_ = 7.2 µM and *n*_*H*_ =  − 0.78.

Therefore, we have shown that GABA_A_Rs in *A. equina* modulate cell excitability in a pH-dependent manner. Equipped with this knowledge, we began to research the impact of GABA_A_Rs on *A. equina* behaviour.

**Fig. 3:**
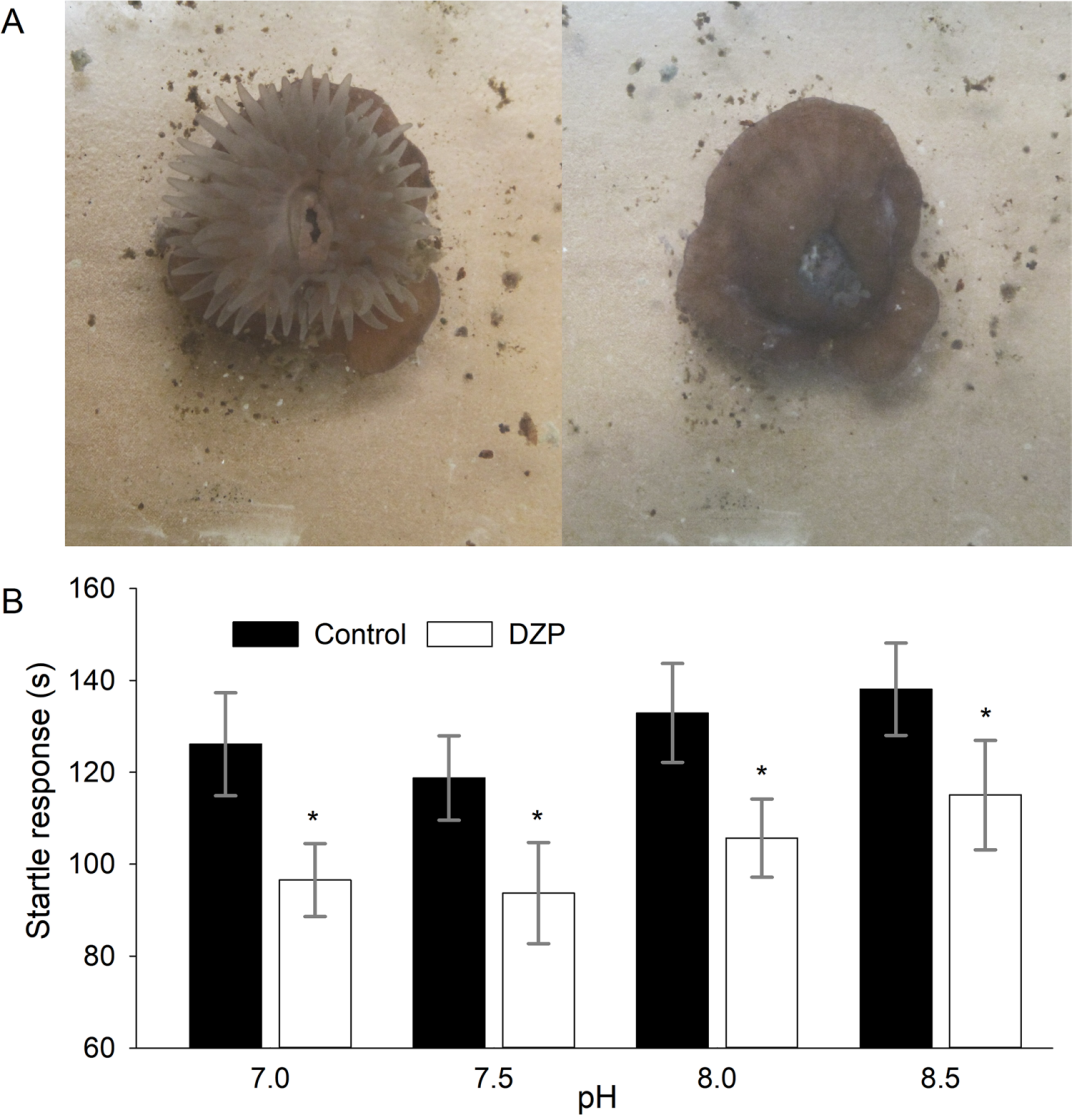
DZP impact on startle response. **A** *A. equina* before (left, open) and after (right, closed) water ejection into mouth opening. **B** Experimental statistics: startle response at different pH. Asterisks denote significance of difference from control at corresponding pH; * *P* < 0.05, Student’s paired *t*-test, *n* = 16 pairs

To do this, we monitored the time length of the startle response (strangulated mouth opening with retracted tentacles, see Fig. 3A) to water ejection into actinia’s mouth. Since DZP added to ejected water alone exerted a significant impact on the response length, we concluded that living animals maintain in tissues a sufficient concentration of GABA to manifest a GABA_A_R-related DZP effect. Here we found that DZP significantly reduces the response length under all pH levels. However, the change of pH itself did not exert a significant effect (Fig. 3B).

RM-ANOVA on the startle response length. Factor 1: *F*_(1, 15)_ = 15.94, *P* = 0.0012; factor 2: *F*_(2.8, 41.95)_ = 1.89, *P* = 0.15; factor 1 × factor 2: *F*_(2.42, 36.48)_ = 0.045, *P* = 0.97. Tukey test on factor 2: *P* > 0.05 for all comparisons.

## Discussion

In this research, we demonstrated for the first time the effect of a specific agonist of a GABA_A_R in Anthozoa at a single-receptor level and then studied the projection of such an effect to modulation of the whole cell excitatory signalling and further to the level of behaviour control. Our data confirm GABA_A_R to be an important factor shaping *A. equina* behaviour via control over AP generation machinery. The absence of DZP effect when the compound is added without GABA in vitro (Figs. 1 and 2) and presence of its effect in vivo (Fig. 3) suggest the continuous secretion of GABA by actinia’s living tissue in a concentration sufficient for activation of GABA_A_Rs.

Despite the principal role of GABA_A_Rs in the delivery of inhibitory signalling being well-established in vertebrates and in a number of invertebrate species, their functional profile in Cnidaria remains unclear. Earlier studies have reported the presence of GABA in different cnidarians [10, 27, 28] and its role as a signalling molecule in feeding behaviour, orientation, tentacle movement, and so on [17, 18, 34]. Pharmacological data suggest the presence of GABA_A_Rs in Cnidaria [33]: an observation that was later supported by genetic evidence [1]. However, the structure of the GABA_A_R derived from the genome of the sea anemone *Nematostella vectensis* [1] differs substantially from that suggested by the pharmacological profile of *Hydra vulgaris* receptors [9, 33], with the latter being similar to vertebrate orthologues. Our data demonstrate the inhibitory effect of DZP, given that it is a specific allosteric agonist of vertebrate GABA_A_Rs*.* However, despite DZP being shown to upregulate the GABA-independent activation of vertebrate GABA_A_Rs previously [3], in our work, we did not observe any significant effect of DZP in the absence of GABA (Fig. 1C). Apart from that and on the contrary to common observations in vertebrates [3], in our recordings from outside-out patches, we failed to register spontaneous GABA_A_R openings (Fig. 1A and C, top traces). Our observations thus give indirect support to the hypothesis of significant structural difference between GABA_A_Rs in vertebrates and in Anthozoa [1].

An important issue arising from the data collected in our work is indeed why fluctuations of pH exert a significant effect on the functional profile of a single GABA_A_R (Fig. 1) and on GABA_A_R-mediated control over AP-generating mechanisms in a single cell (Fig. 2) but not on GABA_A_R-mediated behavioural reactions of living animals (Fig. 3). The low sensitivity of the startle response to GABA_A_R effects is an unlikely reason for this since the startle response experienced a highly significant impact from GABA_A_R-mediated effect of DZP. A plausible explanation is a loss of the GABA_A_R response sensitivity to pH at saturative concentrations of GABA [8, 36] since the protonation of GABA_A_R extracellular domain modulates GABA binding kinetics [30]. To the best of our knowledge, the steady-state concentration of GABA in *A. equina* tissue, as well as the binding constant(s) of GABA_A_Rs subspecies expressed in actinia, has not yet been determined. Therefore, at present we have insufficient data to make a meaningful conclusion as to how close to a saturative level the native GABA concentration maintained in actinia tissues is. However, in vertebrates’ neural tissue, the native concentration of GABA was reported to be of micromolar range [22, 25, 31], which is more than an order of magnitude higher than 100-nM concentration used in our in vitro experiments, where the effect of pH was statistically significant. Additionally, to the best of our knowledge, there are no data on the share of actinia’s GABA_A_Rs localized in synapses vs. at extrasynaptic cell membranes. Taking into account that the concentration of GABA released into synaptic cleft is commonly 3–5 orders of magnitude higher than in extracellular space and far exceeds the level of saturation of GABA_A_Rs [13], a high “synaptic/extrasynaptic” ratio of GABA_A_Rs’ distribution in actinia’s excitable cells may support resistance to pH fluctuations.

By exploring the pH interval from 7.0 to 8.5 in our experiments, we have covered the full range of inter-annual pH fluctuations observed in the North Sea and Black Sea at present (which is 7.5–8.5) [4, 35] plus maximum predicted acidification (pH value decrease by 0.45 unit) [7, 39]). Hence, our data from behavioural tests (Fig. 3) suggest *A. equina* to be well-prepared for any OA scenario: actinia’s nervous system stability and behavioural patterns should not be affected by the predicted pH shift.

## Abbreviations

AP: Action potential
APV: D-2-amino-5-phosphonovalerate
ASAB: Association for study of animal behaviour
CGP-55845: (2*S*)-3-[[(1*S*)-1-(3,4-Dichlorophenyl)ethyl]-amino-2-hydroxypropyl](phenylmethyl)phosphinic acid
DZP: Diazepam
GABA: γ-Aminobutyric acid
GABA_A_R: GABA receptor of type A
GABA_B_R: GABA receptor of type B
MSC: Muscimol
NBQX: 2,3-Dioxo-6-nitro-1,2,3,4-tetrahydrobenzo[*f*]quinoxaline-7-sulfonamide
OA: Ocean acidification
PTX: Picrotoxin
RM-ANOVA: Repeated-measures analysis of variance
